# Un ectropion urétral peut cacher un carcinome!

**DOI:** 10.11604/pamj.2017.27.249.13260

**Published:** 2017-08-03

**Authors:** Mohammed Alae Touzani, Othmane Yddoussalah

**Affiliations:** 1 Université Mohammed V, Faculté de Médecine et de Pharmacie de Rabat, Hopital Ibn Sina, Service d’Urologie B, Rabat, Maroc

**Keywords:** Ectropion, urètre, carcinome épidermoïde, Ectropion, urethra, squamous cell carcinoma

## Abstract

The incidence of female urethral cancer is rare and accounts for 0.02% of all women’s cancers. It is dominated by epidermoid carcinoma, which most commonly develops in the distal portion of the urethra, extending to inguinal lymph nodes. The diagnosis is confirmed in the distal forms on the basis of uretrocystoscopy with biopsy. Abdominopelvic MRI allows to determine tumor extension as well as its infiltration into tissues and peri-urethral organs. As in superficial tumors of the distal urethra, simple circumferential resection of the urethra associated with resection of the adjacent portion of the anterior surface of the vagina is sufficient. We here report the case of a 59-year old diabetic and hypertensive female patient presenting for irritative lower urinary tract symptoms, associated with vulvar mass. Clinical examination showed mucosal ectropion extending from the urethral meatus associated with extensive local inflammation. The patient underwent wide excision of the ectropion which was diagnosed as squamous cell carcinoma. Abdominopelvic MRI was normal.

L'incidence du cancer de l'urètre féminin est rare et représente 0,02% de l'ensemble des cancers de la femme. Il est dominé par le carcinome épidermoïde, qui se développe le plus souvent au dépend de la portion distale de l'urètre, et s'étend aux ganglions inguinaux. Le diagnostic est confirmé pour les formes distales par l'urétrocystoscopie avec biopsie. L'IRM abdominopelvienne permet de préciser l'extension en amont et l'infiltration des tissus et organes péri-urétraux. Ainsi pour les cancers superficiels de l'urètre distal, une excision circonférentielle simple de l'urètre associée à une excision de la portion adjacente de la face antérieure du vagin est suffisante. Nous rapportons ici le cas d'une patiente de 59 ans, diabétique et hypertendue, qui consulte pour des signes du bas appareil urinaire de type irritatif, accompagnés d'une masse vulvaire. À l'examen clinique, il existait un ectropion muqueux accouché par le méat urétral associé à une importante inflammation locale. La patiente a bénéficié d'une exérèse large de l'ectropion qui s'était révélé être un carcinome épidermoïde. L'IRM abdomino-pelvienne faite était sans particularités.

**Figure 1:**
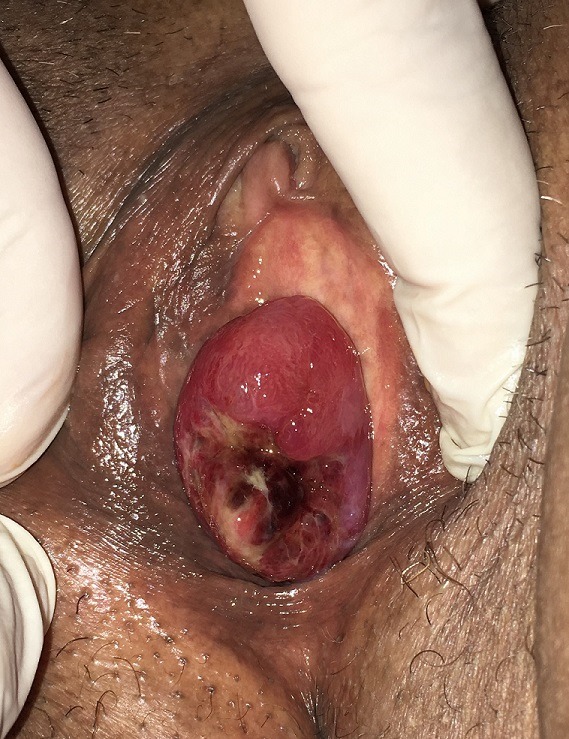
Ectropion

